# Left Main Stem Perforation: Management Strategies in a Potentially Catastrophic Event

**DOI:** 10.1002/ccd.70626

**Published:** 2026-04-13

**Authors:** Hilal Khan, Mohamed Farag, Scott Wilkes, Mohaned Egred

**Affiliations:** ^1^ Cardiothoracic Centre Freeman Hospital Newcastle‐upon‐Tyne UK; ^2^ Department of Clinical, Pharmaceutical and Biological Science University of Hertfordshire Hertfordshire UK; ^3^ School of Medicine University of Sunderland Sunderland UK

**Keywords:** angioplasty, coronary, covered stents, intervention, left, main, perforation, stem

## Abstract

Left main stem coronary perforation is a potentially fatal complication of percutaneous coronary intervention if not recognized early and treated emergently. Treating left main stem perforation can be challenging due to the significant hemodynamic effect of one of the important steps, namely balloon tamponade. Managing such perforation requires speed, precision, and a clear systematic approach to help save lives. We present a case of left main stem perforation that occurred during chronic total occlusion percutaneous coronary intervention which was treated with a covered stent across the left main stem into the left anterior descending artery. Restoration of flow into the circumflex was achieved by using a penetrating wire to cross into the circumflex, achieving hemostasis and preserving coronary flow into both epicardial coronary arteries. We describe further bailout strategies for dealing with left main stem perforations, including left main covered stenting with jailed side branch balloon and the simultaneous kissing stent technique.

## 1 Introduction

Severe left main stem (LMS) coronary artery disease is associated with a high mortality when managed medically [1]. It accounts for 3%−10% of disease in patients undergoing coronary angiography [2]. Traditionally, the mainstay of treatment was with coronary artery bypass grafting (CABG) due to poor results with plain old balloon angioplasty of the LMS associated with a high mortality of up to 64% in early studies [3].

The advent of contemporary drug‐eluting stents (DES) has been shown in several studies to be largely comparable in reducing mortality outcomes when compared to CABG [4, 5, 6, 7]. This has led over time to increasing numbers of LMS disease to be treated by percutaneous coronary intervention (PCI) [8].

The growth of PCI as a treatment option for LMS disease is associated with the occurrence of complications, although rare, but when they do occur, and due to the critical anatomic location of LMS, they are associated with significant mortality and morbidity. The most significant of complications involving LMS is coronary perforation.

LMS perforation occurs in around 0.4%−0.9% of cases treated with PCI [8]. There are unique considerations when using covered stents to treat LMS perforation due to the loss of major side branches, namely the circumflex. In addition to the haemodynamic compromise particularly when tamponading the perforation with a balloon.

Here, we present a case of LMS perforation and strategies for managing these perforations.

## 2 Case Example

**Figure 1:**
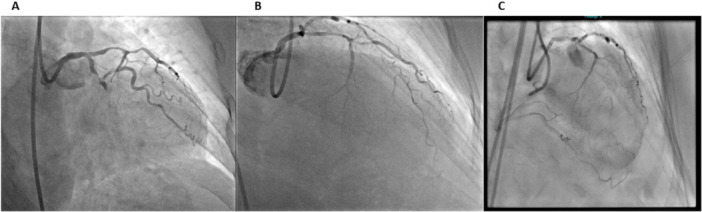
Coronary angiography showing the occluded circumflex (A). Occluded LAD (B). Set up images with bilateral injection showing the occluded LAD with collaterals from the RCA (C).

A 70‐year‐old lady presented for as an elective admission for re‐attempt at PCI to a chronic total occlusion (CTO) of her left anterior descending (LAD) artery. She had a history of non‐ST‐elevation myocardial infarction 3 years earlier when she had PCI to her right coronary artery (RCA) which was calcified and was complicated by coronary perforation at the time and was managed by a covered stent to her RCA (Figure 1).

She also had a CTO of both her LAD and circumflex identified during the index procedure and was brought back previously for revascularisation of these vessels (Figure 1). This attempt was complicated by distal wire perforation from a branch of the posterior descending artery from retrograde wiring and was treated by a coil and covered stent in the main posterior descending artery occluding the perforated side branch. It was decided to pursue medical management for a time, but she was intolerant of nitrates with ongoing angina despite anti‐anginal medications. Stress echocardiogram revealed ischemia and wall motion abnormalities in her LAD territory, otherwise normal left ventricular systolic function, and she agreed and decided to go ahead with another procedure for revascularisation of her LAD CTO by PCI, given the poor quality of the distal vessel rendering it unsuitable for surgery.

The procedure was performed with bi‐femoral vascular access using 6/7 French slender sheaths (Terumo International, Japan). The LMS was engaged with a 7 French Extra Back‐Up 3.5 guide catheter (Medtronic, Minnesota, USA). The RCA was engaged with a 90 cm, 7 French Amplatz Left 1 guide catheter. The distal LAD was wired retrogradely from an apical epicardial collateral arising from the right posterior descending artery using a Caravel microcatheter and Suoh 3 wire.

**Figure 2:**
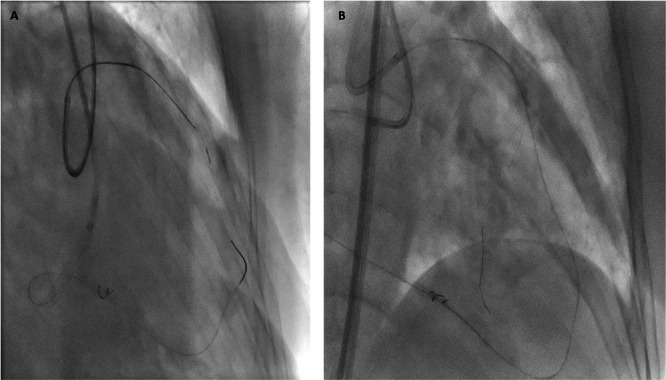
Retrograde crossing with Suoh 3 wire and Caravel microcatheter and antegrade with Corsair Pro and Gladius EX wire (A). RG3 wire exchanged with retrograde wire and externalized (B).

Antegrade, the LAD was wired with a Corsair Pro microcatheter and a Gaia Next 3 wire (Asahi Intec, Japan) to puncture the proximal cap and advanced a Gladius EX wire (Asahi Intec, Japan) into a septal branch antegrade (Figure 2). Then a Gladius MG wire (Asahi Intec, Japan) was retrogradely advanced into the antegrade guide catheter, and the wire was exchanged for an RG3 wire (Asahi Intec, Japan) using the microcatheter (Figure 2). Predilatation was performed with a non‐compliant balloon, then the LAD was wired antegrade and the retrograde gear removed. Two overlapping DES were deployed in the LAD back to its proximal part.

**Figure 3:**
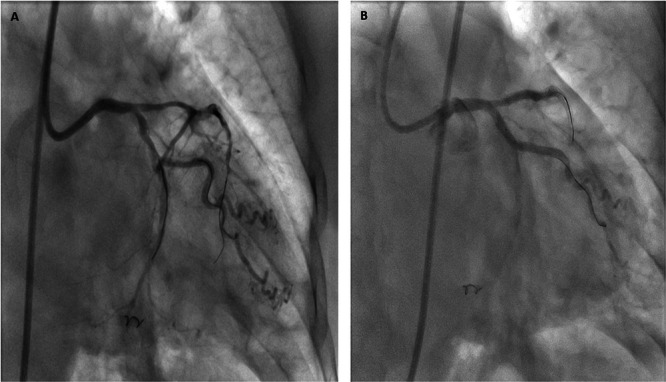
Circumflex chronic total occlusion revascularized (A). Results in circumflex after stenting from the obtuse marginal to the LMS (B).

Following on, the circumflex CTO was traversed with a Gladius MG wire and predilatation performed (Figure 3) after which the obtuse marginal branch was wired with a Fielder FC (Asahi Intec, Japan) and predilated, then stented back to the LMS with two overlapping stents (Figure 3). Following on, the LAD was recrossed, then a DES was inserted from LMS into LAD.

**Figure 4:**
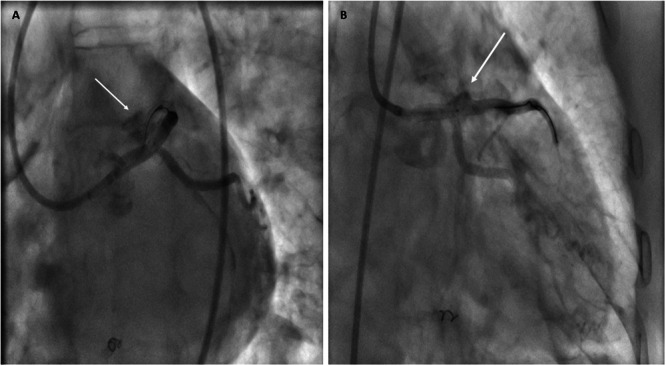
Evidence of extravasation and perforation of LMS (white arrows), in steep caudal view (A), and in a straight caudal view (B).

**Figure 5:**
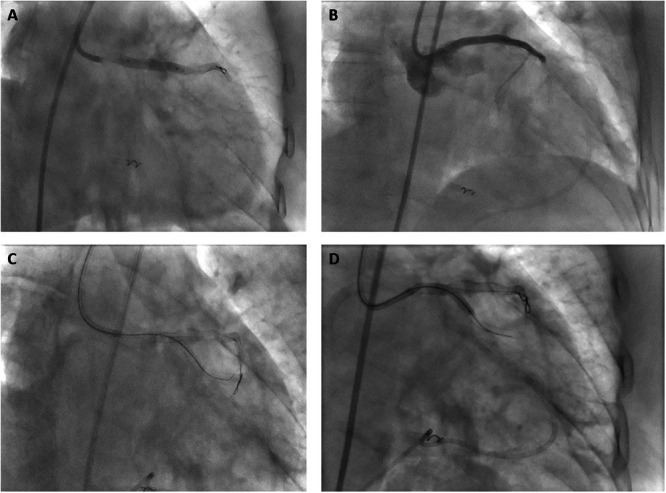
Balloon tamponade of the left main stem with no flow (A). Occluded circumflex after covered stenting of the left main stem (B). Sasuke dual lumen microcatheter and Confienza pro 12 used to pierce covered stent into circumflex (C). Circumflex ballooned to restore flow (D).

After deployment of the LMS stent, there was evidence of LMS perforation and extravasation (Figure 4) with haemodynamic compromise with evidence of contrast in the pericardium. Prompt balloon tamponade was carried out, and pericardiocentesis was performed (Figure 5). Two covered stents (PK Papyrus, Biotronik, Germany) were deployed, a 4.0 × 15 mm and a 5.0 × 15 mm from the LAD and overlapping back into the LMS.

**Figure 6:**
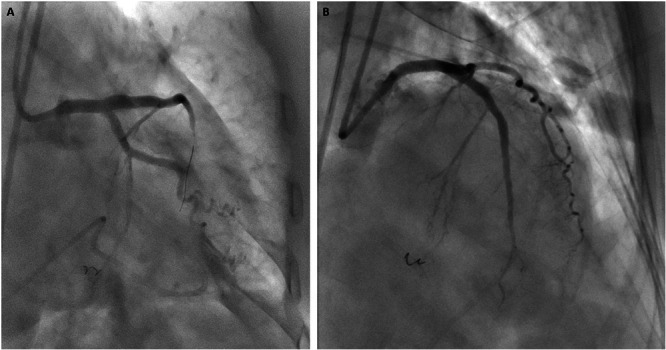
Final angiogram views. Patent circumflex (A) and left anterior descending artery territories (B) show no more extravasation.

A Sasuke dual lumen microcatheter (Asahi Intec, Japan) was used to deliver a Confianza Pro 12 wire (Asahi Intec, Japan) and puncture through the covered stent into the circumflex with subsequent sequential ballooning re‐establishing flow to the circumflex (Figures 5 and 6). The LMS was then post‐dilated with a 5.0 × 15 mm balloon with excellent results and no evidence of extravasation.

During the night, the patient's blood pressure was recorded to be low, and an echocardiogram showed a pericardial collection. The on‐call cardiologist interpreted this as loculated and clotted fluids that would be difficult to drain percutaneously. He contacted the cardiac surgeon, and the patient had a surgical sternotomy and fluids removed. This was congealed, and serous fluid, and there was no ongoing bleed noticed during the sternotomy. The patient made a complete recovery and was discharged home in a stable condition and remains well.

## 3 Discussion

LMS perforations are a potentially catastrophic and fatal complication. The importance of recognizing high‐risk features for LMS perforation, early recognition of perforation when it occurs, and the ability to manage it effectively by achieving hemostasis and maintaining blood flow in both epicardial coronary arteries are key to achieving successful percutaneous treatment.

### 3.1 High Risk Features for LMS Perforation

Several risk factors are associated with the risk for coronary perforation in general. These could be broadly delineated into: (a) patient characteristics, (b) coronary anatomy, and (c) procedural features (Table 1) [8, 9].

**Table 1:** Risk factors for coronary perforation during percutaneous coronary intervention.

Risk factors for coronary perforation	
Patient factors	Procedural/Anatomic factors
Increasing age	Previous coronary artery bypass grafts
Female gender	Chronic total occlusion
Hypercholesterolemia	Left main stem stenting
Presentation with non‐ST‐elevation myocardial infarction	Multiple stent usage
	Calcified coronary artery disease
	Rotablation usage

### 3.2 Early Recognition and Management of Coronary Perforation

It is of paramount importance to anticipate cases at higher risk of coronary perforation, which makes it easier to identify coronary perforation early when it does occur, which in turn is vital in allowing successful management. This can usually be achieved by a small contrast test, particularly with calcium, and after high‐pressure ballooning. Sharp chest pain during balloon or stent deployment should raise suspicion of coronary perforation [10]. Coronary perforation can be in the large epicardial vessel, in the small distal vessel, and in collaterals. The severity of coronary perforation is assessed using the Ellis classification. Class 1 perforations can usually be managed conservatively or with balloon tamponade if required. Class 2 perforations typically warrant some period of balloon tamponade for 10−15 min. Class 3 perforations, however, require emergent treatment with balloon tamponade and covered stenting [11].

### 3.3 Management Strategies for LMS Coronary Perforation

When dealing with LMS perforations, especially those involving the distal LMS, the management is more involved and complex as it requires immediate balloon tamponade and covered stenting across the circumflex which will occlude this major epicardial coronary branch, and in a patient already unwell and suffering from the effects of coronary perforation and tamponade, this will not be well tolerated if left untreated.

It is important to develop strategies for treating this potentially catastrophic complication.

We would advocate three approaches to treating LMS perforation.

#### 3.3.1 Covered Stenting of LMS and Fenestration of Covered Stent into the Circumflex Using Penetrating Wire

The preferred technique would be to stent the LMS with a covered stent jailing the circumflex. Then a dual lumen or angulated microcatheter if more support is needed and a penetrating wire such as a Confianza pro 12 or Gaia Next 3 can be used to penetrate the covered stent to restore flow into the circumflex [12]. The presence of markers of the circumflex before piercing the covered stent with a penetrating wire would be helpful. If there is a previous stent or calcium at the ostium of the circumflex, this can be used as the marker. If, however, neither is present, then leaving a wire to mark the circumflex can be used before deploying the covered stent [13].

It is important that sequential ballooning through the covered stent is performed to prevent damage to its structure. Initial ballooning with 1.5, 2.0 balloons to restore circumflex flow without damaging the overall integrity of the covered stent is vital [14].

#### 3.3.2 Covered Stenting of LMS and Jailed Balloon in Side Branch

Another method which can be used to restore flow would be to ensure there is a wire and a small 1.5 mm balloon in the circumflex before deploying a covered stent in the LMS which jails the circumflex. The proximal edge of the balloon should be proximal to the proximal edge of the covered stent and the distal edge should be in the proximal circumflex and a balloon sufficiently long to achieve this should be selected. The covered stent is first deployed, and this is followed by inflation of the jailed side branch balloon, restoring flow to the circumflex while achieving hemostasis [15, 16]. This method may not be as effective and may deform the covered stent with reduced flow into the circumflex due to the limited size of the balloon that can be used for this method.

#### 3.3.3 Left Main Covered Stenting With Kissing Stent Technique

A last resort option if one is unable to perform either of the two approaches detailed above would be to deploy two covered stents from LMS, one into the LAD and one into the circumflex as a kissing stent technique which will achieve hemostasis as well as maintaining flow in both epicardial coronary arteries [17]. Alternatively, a covered stent and a DES can be used simultaneously instead of two covered stents, if the site of the perforation is deemed suitable.

There is limited data on V‐stenting techniques (when the perforation is deemed to be at the ostial LAD or circumflex), but they seem to be associated with high early re‐stenosis rates [18]. Furthermore, with a new carina created, future intervention may be difficult; however, this method should be reserved for extreme cases if other methods are not feasible.

Obviously, in this method, a large guide catheter (8 French) is required to allow for the accommodation and delivery of the bulky, large‐sized covered stents. Alternatively, using two guide catheters (Ping Pong technique) is required for the delivery of two simultaneous covered stents, or one covered and one DES.

Irrespective of which strategy is adopted to manage LMS perforation, action should be enacted speedily and in a timely manner due to the catastrophic nature of LMS perforations and the imminent hemodynamic compromise that develops very quickly with balloon tamponade.

In the author's practice, a DES (longer than the intended covered stent) is deployed first, followed by the covered stent inside it. The rationale for this is to reduce the rate of restenosis with the abluminal drug on the DES in contact with the arterial wall. There is no data to support this practice, but it has been the approach in the senior author's practice for many years, with very few cases of restenosis. Other operators chose to deploy the covered stent, then follow with a DES inside. There is no data to support either approach.

## 4 Conclusion

LMS perforation is a rare occurrence and can be catastrophic. It is important to recognize the risk factors for coronary perforation and to recognize the perforation early when it does occur. Three techniques for managing this complication can be used. First, LMS covered stent and fenestration of the covered stent into the circumflex using a penetrating wire. Second, LMS covered stenting with a jailed balloon in the circumflex to maintain circumflex patency. Third, the simultaneous kissing stents technique with a covered stent in the offending branch and DES in the side branch to maintain patency of both epicardial coronary arteries.

## Funding

The authors have nothing to report.

## Disclosure

The authors have nothing to report.

## Conflicts of Interest

The authors declare no conflicts of interest.

## Data Availability

The data that support the findings of this study are available from the corresponding author upon reasonable request.
